# Strategical Pedagogy for the Development of Socio-Emotional Competences in Nursing Students

**DOI:** 10.3390/nursrep14040208

**Published:** 2024-10-08

**Authors:** Laura Andrian Leal, Carolina Cassiano, Paulo Cruchinho, Elisabete Nunes, Pedro Lucas, Gisela Teixeira, Silvia Helena Henriques

**Affiliations:** 1 Departament of Hospitalar Nursing, University Federal do Triângulo Mineiro, Uberaba 38025-180, MG, Brazil; 2 Nursing Research Innovation and Development Centre of Lisbon (CIDNUR), Escola Superior de Enfermagem de Lisboa, 1600-190 Lisboa, Portugal; pjcruchinho@esel.pt (P.C.); enunes@esel.pt (E.N.); prlucas@esel.pt (P.L.); gteixeira@esel.pt (G.T.); 3 School of Nursing of Ribeirão Preto, University of São Paulo, São Paulo 14040-902, SP, Brazil; carolinacassiano03@gmail.com (C.C.); shcamelo@eerp.usp.br (S.H.H.)

**Keywords:** education, nursing, emotions, health services, professional competence, social skills, students, nursing

## Abstract

Socio-emotional competencies (SECs) are essential for the quality of nursing practice. This study aimed to understand the pedagogical strategies provided by universities for the development of SECs in Brazil, as perceived by nursing students. This is a descriptive, exploratory study with a qualitative approach involving 57 nursing students from a public Higher Education Institution in Brazil. Data collection was conducted through focus groups, and interpretation was carried out using inductive thematic analysis. The pedagogical strategies identified were categorized as follows: those offered by the curriculum matrix, those provided by the Nursing course department, those made available by the university as a whole (involving all courses and students), and the challenges related to the development of socio-emotional competencies. This study revealed a deficit in the integration of SECs in the curriculum, highlighting the need for curricular restructuring and discussions on this topic. Equipping students with socio-emotional competencies during their training can help address these gaps, fostering greater resilience and self-care capabilities among nursing professionals.

## 1. Introduction

In recent years, global technological and educational advancements have underscored the importance of developing pedagogical strategies that promote changes in the training and professional practice of nurses. Consequently, nursing education has shifted toward a competency-based approach to support qualified health care practice [1,2].

The concept of professional competence has drawn attention from both nurses and health managers. Nationally, professional competence can be defined as the integration of behaviors that ensure effective work outcomes through the application of knowledge, skills, and attitudes that support high-quality professional performance [3].

Among these competencies, socio-emotional competencies (SECs) have increasingly garnered attention in higher education [4,5,6,7]. Internationally, these are also referred to as “non-cognitive competencies”, “21st-century competencies”, “character strengths”, or “emotional intelligence” [8]. SECs can be defined as individual characteristics that (1) link biological predispositions with environmental factors, (2) are expressed through consistent patterns of thought, feeling, and behavior, (3) are developed through both formal and informal learning experiences, and (4) influence socio-economic outcomes throughout life [9]. These competencies include interpersonal skills, such as teamwork, and systemic competencies, such as leadership abilities [10]. Within the educational process, SECs can be considered both as a means and an end [11].

Internationally, SECs play a crucial role in promoting professional skills among nurses, fostering both their personal development and improving interactions with patients and their families. These competencies allow nurses to create an empathetic and supportive environment, enhancing trust and collaboration between patients and caregivers. Thus, socio-emotional competence is essential to nursing practice [11,12,13,14,15,16].

Socio-emotional competencies, internationally classified as emotional intelligence (EI) competencies [12], are associated with the ability to apply emotional intelligence concepts successfully in daily practice. This includes effectively leading and influencing individuals and groups by utilizing attributes such as self-awareness, humility, resilience, and optimism. Like other competencies, SECs can be developed over time [13,14]. The literature suggests that affective and relational competencies are among the most crucial areas for improvement, especially since nurses face challenges such as exhaustion, multitasking, and adherence to institutional norms. Researchers have identified SECs, such as ethics, listening, patience, and relationship building, as those most in need of development within the hospital sector. This was highlighted through a Likert-type questionnaire administered to nurses in a Brazilian hospital, followed by descriptive statistics and Mann-Whitney U tests [1].

SECs also carry cultural significance, as they are closely linked to how individuals from various cultures perceive and express emotions, resolve conflicts, and interact socially. Different societies shape expectations for how these skills should be developed and expressed. For instance, collectivistic cultures, such as those in Asia, may emphasize socio-emotional skills that promote harmony and cooperation, whereas individualistic cultures, such as those in the United States and many Western countries, may prioritize skills like assertiveness and autonomy. The transferability of SECs across cultures is a complex issue. Although some competencies, such as empathy or conflict resolution, may apply broadly, how they are practiced and perceived can vary significantly. Therefore, cultural adaptation must be considered when discussing SECs’ transferability. Training programs that are designed from a single cultural perspective may struggle to be effective in diverse contexts. To ensure the effectiveness of SECs in international nursing practice, a focus on cultural sensitivity and pedagogical flexibility is required [7,11,12,13,14].

A recent international scoping review aimed at mapping SEC development strategies in nursing schools identified various methods, including simulation-based learning, social skills camp programs, emotional intelligence models, learning through escape rooms, blended learning, debate as a strategy, action-based learning, and courses on grooming and etiquette [15]. The review found that the five main competencies developed through these strategies were communication, teamwork, critical thinking, confidence, and situational awareness [15]. Additionally, a systematic review by Jamaludin et al. [16] concluded that, without the development of SECs in clinical education, graduate students are often unprepared for professional careers, recommending the integration of SEC elements into student evaluation processes [16].

Thus, it is observed that there are few descriptions of SEC development strategies needed for the training of nursing students for future work in the international nursing field [1,15,16]. In this context, SECs are necessary for the improvement of nursing graduates and the profession itself after their training to positively influence the work environment [14].

SECs are recognized as important for the nursing practice, but the production of detailed academic and pedagogical literature on the topic still faces obstacles. The inherent complexity of these skills, difficulties in measuring and evaluating them, cultural barriers, and the traditional focus on technical skills limit the development of studies that address the dynamics of socio-emotional skills in a pedagogical and structured way. To advance in this area, a joint effort is needed to integrate these skills into the nursing curriculum, invest in research that demonstrates their importance, and promote the greater pedagogical appreciation of socio-emotional skills within professional training [1,8,9,10,11,12,13,14,15,16].

Therefore, for some international researchers, the main pedagogical strategies for competency development involve creating specific spaces such as integrating SECs into technical subjects, using active methodologies, and developing cross-cutting activities throughout the training. It is emphasized that pedagogical strategies refer to planned methods and approaches that educators use to facilitate learning and the development of specific skills in students [7,8,9,17].

Moreover, in education, several pedagogical strategies have had to be modified to continue promoting meaningful learning for nursing students using technological resources, computerized systems, educational videos, and remote simulations [18,19]. In this sense, ‘meaningful learning’, ‘successful careers’, or ‘transformative learning’ refer to contexts in which new knowledge connects in a relevant and logical way to the individual’s previous knowledge. Instead of rote memorization, meaningful learning integrates new information deeply, allowing the student to understand and apply the content in different contexts. This process promotes a more lasting and applicable understanding, as knowledge becomes part of an organized and functional cognitive structure [4,5,6].

From this perspective, SEC-based teaching can act as a strategy to face the transformations occurring in the work world, particularly aspects related to emotions and mental health.

To address SECs, it is essential to rethink curricular changes, pre-existing trust relationships established with students, and collaborative systems that support quick responses. Joint problem solving and a harmonious environment contribute to the development of successful strategies and robust processes that help rethink and prepare professionals to deal with emotions and interactions, as noted by local researchers [20].

In this sense, considering the various assignments and care provided by nurses, along with the intensifications in work processes due to the pandemic, it becomes essential to discuss SEC for nurses and their development strategies. These strategies can provide essential aspects for equipping the work of nurses and students in care, as well as offering elements to perform nursing care more safely, sensitively, and creatively.

This study addresses the following guiding question: What strategies has the university implemented for developing SECs in nursing students in Brazil? Describing the pedagogical strategies for developing SECs is essential due to the critical need to prepare nurses not only with technical knowledge, but also with socio-emotional skills such as empathy, resilience, and effective communication. Traditional nursing education often neglects these competencies, leaving professionals less prepared to face the emotional and social challenges of the profession [1,4]. By exploring and implementing pedagogical strategies, it is possible to promote more humanized and effective professional practice, improving patient care quality and nurses’ well-being.

Therefore, this study aimed to understand the pedagogical strategies provided by the university for the development of SECs in Brazil, as perceived by nursing students.

## 2. Materials and Methods

### 2.1. Type of Study

This is a descriptive exploratory study using a qualitative data approach. This study aims to understand complex phenomena in depth, exploring participants’ perceptions, behaviors, and experiences without trying to quantify results. This type of study is characterized by the collection of non-numerical data, such as interviews, focus groups, and observations, and seeks to generate insights and initial hypotheses on a topic that is still little studied or for which there is little prior knowledge. The focus is on obtaining a detailed and contextualized understanding, valuing the nuances and particularities of the data collected. Furthermore, this investigation was conducted using the Consolidated Criteria for Reporting Qualitative Research (COREQ) guide from the Equator Network [21].

### 2.2. Setting of Study

The study was conducted at a Brazilian public Higher Education Institution (HEI) in a municipality in the interior of São Paulo. The HEI offered two types of courses: “Bachelor’s” and “ Bachelor’s and Degree”. Both courses certify about 130 admissions annually, with 80 students for the Bachelor of Nursing course and 50 students for the Associate and Bachelor in Nursing course. It is noteworthy that, in this institution, the Associate course lasts four years with eight semesters, while the Associate and Bachelor course lasts five years with ten semesters.

### 2.3. Population and Selection Criteria

Study participants were final-year undergraduate students who were taking or had completed the Hospital Nursing Management subject. These students were chosen due to their greater knowledge, having completed management and administration internships (which address SECs), and having had the experience of learning and approaching the SECs necessary for nursing practice. Students with locked enrollment or who were away were excluded from the study.

This educational institution was selected because it is a pioneer in nursing education in the country researched, and due to the connection of the researcher involved in data collection.

### 2.4. Period of Study

Data collection was conducted from March to September 2023.

### 2.5. Data Collection

Data were collected through focus groups (FGs) with students from the selected HEI. Regarding participant selection techniques for data collection, intentional non-probability sampling based on judgment was used. Furthermore, the Snowball sampling technique was employed involving the target audience, which consisted of nursing students. Participants were formally invited in person by letter (after class) or electronically to schedule the research activity according to their availability.

The FGs were conducted randomly, all in person, and were carried out according to the offering of the Hospital Management subject by different classes and courses.

The FGs followed Gatti’s [22] methodological framework and the research’s guiding questions were led by the researcher (moderator) and a research assistant (observer). Initially, a sociodemographic data questionnaire was administered, followed by the FGs using a guide that contextualized SECs to provide direction for participants. This included guiding questions on the topic, which were validated and tested by experts. The guiding questions were as follows: (1) What are the pedagogical strategies that you think the university uses to develop SECs in the Nursing course? (2) Do you notice if there are differences in the strategies offered by the course matrix, the department, and the university in general? Discuss each. (3) How do you nursing students perceive the effectiveness of these strategies in developing SECs for practice? (4) What challenges do you face in implementing pedagogical strategies for SECs? All sessions were recorded and scheduled according to participants’ availability.

Each FG was conducted in accordance with the guide and questions previously explained. To clarify, each FG followed these steps. First step: Period of awareness, selection, and assembly of the FGs (participants were selected into smaller groups, as recommended by the theoretical framework, a field diary was prepared for group discussion notes, and the FGs were organized based on participant availability).

Second step: Development of the FGs by organizing the environment (arranging groups in a room with an oval table for better interaction, providing a comfortable and ventilated environment with coffee and sweets, and offering paper and pens for note taking).

Third step: Conducting the FGs (introduction of the moderator and observer, followed by a presentation and definition of the topic, establishing a time contract, anonymity, and using the guiding questions from the guide) [22].

### 2.6. Data Analysis

The discussions from the FGs were manually transcribed and interpreted using inductive thematic analysis, following these steps: transcription and reading of data; systematically coding interesting features across the entire dataset; searching for themes by grouping codes; reviewing and verifying the themes; refining the details of each theme; and conducting a final analysis of selected excerpts related to the research and literature questions, generating an academic report of the analysis [23].

The analysis phase was carried out by the lead researcher, who was experienced and trained in the aforementioned method, contributing to the reflexivity of the findings. Furthermore, the analysis was carefully reviewed and validated by the other authors of this study.

### 2.7. Trustworthiness

To ensure the trustworthiness of the study, some strategies described by Ahmed [24] were followed. Credibility was demonstrated by the number of participants, the number of focus group interviews conducted with different students, and the number of data collection methods combined in this study. Transferability was confirmed by the density of contextual data described. Dependability was evidenced by the degree of detail of the methodological aspects followed in the study. Confirmability was ensured by validating interpretations with two members of the research team [24].

### 2.8. Ethical Considerations

This study adhered to Resolution 466/12, approved by the Research Ethics Committee of the Proposing Institution (Opinion No. 5.803.350 of 2022). Participants signed the Informed Consent Form, ensuring the confidentiality of their responses.

## 3. Results

Of the 110 students invited, 57 (51.81%) participated, with 51 (89.47%) being female and 6 (10.52%) male. Ages ranged from 20 to 31 years, with an average of 23.01 years. Regarding the course, 41 (71.92%) were students from the Associate Nursing course, with 20 (48.78%) in the 7th semester and 21 (51.21%) in the 8th semester. In the Associate and Bachelor course, 16 (28.07%) students participated, with 13 (81.25%) in the 9th semester and three (18.75%) in the 10th semester.

Nine FGs were conducted. During these sessions, care was taken to ensure the environment was prepared with mild aromas, proper ventilation (via air conditioning or open windows), and lighting. Efforts were made to maintain privacy while providing a cozy atmosphere, including water and snacks available at the end of meetings. Relaxing music was played to create a comfortable environment. The warm welcome of participants was a priority for the coordinators, ensuring a smooth process as participants were located within the institution itself.

Regarding pedagogical strategies for developing these competencies, the following categories were identified: strategies offered by the curriculum matrix, the Nursing Department, the university as a whole, and challenges related to developing SECs, as evidenced in Table 1 and demonstrated through participants’ quotes.

### 3.1. Offered by the Curriculum Matrix

Gaps were identified concerning forms for developing SECs through the curriculum matrix. Among the few evidenced strategies, these were more present in the Bachelor’s scope compared to the Associate’s. Students pointed out that some subjects focus on SECs related to assertive communication and the student’s professional posture. In the case of the Bachelor course, more strategies were observed, focusing on the future teacher’s development and foundation on how to behave in the classroom:

*“I think because it’s part of the Bachelor course, our course brings more about the posture of the registered nurse or even the teacher in how this relationship between teacher and student would be and what the best didactics would be, etc. So, in that sense, what I perceive is that in subjects focused on education, it is discussed about the teacher’s posture, communication methods, different ways of giving classes to improve the bond, and all that we can apply in patient care. So, in this part, yes… in classes more focused on health, this side is not well developed”* [FG 6. Student 4].

*“I think we in the Bachelor are more privileged. I don’t know how it is worked in the Associate’s, but here in the Bachelor, the education subjects have a lot of this about autonomy, non-violent language, and mainly in the classroom, we can extrapolate this to care, getting to know the student-patient, and there are health-focused subjects that also have this active listening in the first years”* [FG 5. Student 10].

*“I am in the Bachelor course, and in the Bachelor, we had a career planning course, which was nice because it addressed how to behave at work involving conflicts”* [FG 5. Student 5].

*“I think here in the Associate’s in nursing management, it is the first subject that talks more about the socio-emotional side, bringing about posture as an important aspect and in simulations, but this only appeared now in the last year”* [FG 8. Student 6].

Some subjects enable the development of SECs through simulations, discussion circles, and case discussions, which stimulates the development of SECs:

*“I think even though there are very few opportunities, we also have case discussion circles, internship groups, and simulations where we learn a bit of the socio-emotional side, but simulations are more practical procedures; there is nothing more focused on professional posture, you know”* [FG 2. Student 4].

*“In the management subject, there are many simulations focused on managerial issues and the socio-emotional side. For example, conflict management enables us to learn how to behave, how to deal with difficult people, or even how to be the ideal professional (with empathy, assertive communication, flexibility, among others…)”* [FG 1. Student 1].

*“The management internship group itself, because we are separated into groups of eight per teacher, helps discuss cases related to socio-emotional aspects, we share experiences”* [FG 3. Student 2].

The support of model professionals, such as nurses and other field professionals from the internship settings, was observed as important for developing these competencies in terms of non-violent communication and welcoming. There are subjects that stimulate feedback and emotional intelligence. Additionally, at the selected HEI, there are nurses hired to support clinical placement, who are not teachers but assist in the subject:

*“There was this death in the child health subject, the next day, the teacher came to talk to me and my partner, but she came to me and said: ‘Oh, I heard what happened yesterday. Do you need to talk? How are you feeling about this?’ So, it was a very positive thing that I saw, I use this teacher as a model, I felt her openness in the situation I experienced”* [FG 7. Student 5].

*“One thing I find cool is the presence of school support nurses in internships because they end up being not better, but they know how to handle the situation better than the teacher in explaining what is happening, giving a ‘scolding’. Sometimes it feels like the teacher looks at you from above with superiority, and some nurses I experienced treated you equally, and it was okay not knowing how to do it at that moment because she was with you, you know. So, it was very cool”* [FG 5. Student 3].

*“But what I would praise in this sense is that after all the simulations we had, we had debriefing moments, we had feedback from teachers and nurses, and this is already a development of how our posture should be in these competencies we are discussing here… so I use the example of teachers and nurses from school to have this competence”* [FG 4. Student 2].

### 3.2. Offered by the Department

There were statements about strategies not necessarily part of the curriculum matrix, meaning not all students had this opportunity, and these are specific aspects of the department, such as the following extracurricular activities: student association, tutoring, scientific initiation projects, or workshops linked to post-graduation.

*“We had a CBT strategy [Cognitive Behavioral Therapy] offered by a doctoral student to understand our emotional side and other issues like family distance or dealing with distances during graduation, we talked in groups about this and developed that skill together, so sometimes there was an exercise for us to do, like writing this, that… and it really had teaching more about how to learn these skills. The doctoral nursing student accompanied us for three months, and after some time, she came back to talk to us again to see how we were doing, if we were managing to use what was worked on, but it was the only time in the entire graduation that I had this, it was one semester of college”* [FG 3. Student 1].

*“We had tutoring in our first year of graduation, where we could talk about career planning, we have student welcome associations, research groups working from this perspective, it depends on the tutor and supervisor of the research. In the PET [Tutorial Education Program] there are courses to develop conflict skills and management in discussion circles, and about suicide groups”* [FG 2. Student 1].

*“That moment now with the group, which is from the post [graduation] is helping to develop emotional competence, but this is not mandatory in graduation”* [FG 6. Student 1].

### 3.3. Offered by the University

In addition to the strategies within the curriculum and department, the university offers resources that support the development of SECs, such as gym access, swimming, and psychological services:

*“USP encourages physical activity, nutrition, and socializing, which is great. While USP demands a lot from us, it also recognizes the need for emotional well-being. We have access to a gym, swimming, and psychologists, although it can be difficult to get an appointment. The mental health support is there, even though we still struggle with the pressure”* [FG 9. Student 6].

*“We have a gym and swimming, which helps develop socio-emotional skills because it encourages us to reconnect with ourselves and gain self-awareness”* [FG 3. Student 2].

### 3.4. Challenges

Participants revealed that, despite strategies to develop SECs, challenges remain at the university, particularly a lack of support from the department for incorporating these skills into the curriculum. Many participants expressed that the curriculum matrix lacks strategies for professional development, and teacher interactions sometimes hinder SEC development, with more emphasis placed on patient interaction than on professional training itself.

*“Much is said only about the patient, but for professionals, it is difficult”* [FG 1. Student 2].

*“Look, honestly, I think it’s very superficial, I think tt’s talked about a lot, but little is put into practice because, I speak out as a beginner, we learn more about how to deal with and behave with the patient, but nothing focused on our own development as professionals and as a team”* [FG 2. Student 4].

*“Nothing practical, in fact, there was not even a class, we didn’t have a class on this, we were told to do a work on it”* [FG 3. Student 4].

*“I felt that the subject [psychology applied to health] was more about learning about patient diversity, giving this look to them, but not using it with us, we learned only about mourning, denial, other questions”* [FG 4. Student 2].

Moreover, other challenges listed were the lack of teaching didactics, unpreparedness, lack of teacher welcoming, and methodological updates as important aspects visualized by students when trying to acquire SECs:

*“Most teachers are tyrants, and those who are not are excluded or ridiculed by other teachers, so sometimes it is not well regarded by those who are already there. There are also teachers who welcome but have terrible didactics, do not know how to teach, and when the teacher comes to talk to you, you already withdraw due to their rigid posture. And there are few others who welcome you, I am one of the few who had the luck to be welcomed because the rest had a bad experience, were afraid of being alone, etc., besides the stories we already know from the veterans about bad experiences”* [FG 5. Student 6].

*“Few teachers provide feedback, but at least we have access to a bond with other professionals that help develop these competencies”* [FG 6. Student 3].

*“Old teachers do not update themselves, continue with the same way of teaching as 30 years ago, and things have changed, we are in the era of technology, but they do not see this socio-emotional side”* [FG 4. Student 3].

## 4. Discussion

The results allowed us to verify that pedagogical strategies are associated with a few components present in the curriculum matrix offered by the Nursing Department and the university as a whole. Moreover, challenges associated with these strategies for developing SECs were evidenced.

The absence of subjects focused on developing SECs in the undergraduate nursing curriculum is a complex and multifaceted issue that can be analyzed from different perspectives. Firstly, it is necessary to understand the historical context and traditional structure of Nursing courses. Historically, nursing education has focused on providing the technical and scientific knowledge necessary for health care, meeting the practical and immediate demands of professional practice. This emphasis on technical competencies is understandable, considering the critical nature of nursing work, which often deals with emergency and high-complexity situations.

However, this technical and biomedical approach may overlook the importance of SECs, which are fundamental for a holistic and humanized nursing practice. Competencies such as empathy, communication, conflict management, resilience, and emotional intelligence are essential for effective interaction with patients, families, and multidisciplinary teams. These skills significantly contribute to the quality of care, patient satisfaction, and the well-being of nursing professionals themselves [7,25].

The integration of SECs into nursing education would require an innovative curricular approach that combines theory and practice in an integrated manner. This can include specific subjects but also incorporate these competencies into existing modules through active learning methodologies, such as simulations, role playing, and guided reflections on clinical experiences, as seen in our findings [8,25].

In research development, we observed that the group–researcher device itself and the sociopoetic approach constituted a strategy for valuing the socio-emotional approach with students, provoking the mobilization of SECs among participants. This shows that simple devices requiring little time can be adopted in undergraduate courses to create processes for developing SECs [14,25].

Moreover, the Bachelor’s course was identified as having the most strategies in its curriculum matrix, which may be associated with the educational focus of the course. Additionally, Bachelor’s courses often incorporate more pedagogy and educational psychology subjects that directly address the development of interpersonal and communication skills. These subjects teach strategies for creating positive and inclusive learning environments, facilitating SEC development.

In this sense, it is important to emphasize that, regardless of the course modality, the legal exercise of nursing involves education and people management, which requires SECs. Therefore, it is essential to reflect and stimulate discussions that promote curricular reforms. The National Curriculum Guidelines for the Nursing course have already established a competency-based curriculum focused on ethical, political, socio-educational, and management areas. However, instituting SECs is known to be a slow and gradual process [9,26].

Additionally, the curriculum matrix, by incorporating good professionals in clinical teaching, enables meaningful learning, as seen in our findings. Learning through exemplary professionals, also known as imitation or observational learning, is a pedagogical approach where students develop skills and competencies by observing and following the example of experienced and successful professionals in their field. This method is highly valued in professional and technical education, including nursing, due to its various benefits. By adopting professional models or examples, students have the opportunity to observe everyday practices and approaches that exemplary professionals use to solve problems and make decisions [27].

Another category observed in the findings was the pedagogical strategies offered by the Nursing Department. Similarly, researchers evidenced that the use of strategies such as student associations (an initiative present in universities that provides students with the opportunity to expand their knowledge in specific areas of higher education), tutoring, and research projects plays a fundamental role in developing socio-emotional competencies in nursing students. These educational approaches go beyond traditional teaching, promoting a more dynamic and collaborative learning environment essential for training nurses capable of dealing with the complex challenges of professional practice [15,28].

Engaging in research activities requires a deep commitment to critical thinking and problem solving. During the research process, individuals are constantly challenged to analyze data, formulate hypotheses, test their ideas, and interpret results [7,29]. This continuous practice enhances the ability to think critically and approach problems in a systematic and logical manner, skills valuable for acquiring SECs [18].

By participating in extension activities, such as those promoted by academic leagues, students have the opportunity to engage with the community, and to better understand the needs and realities of the population they will serve. This promotes empathy, cultural sensitivity, and a commitment to improving public health.

Furthermore, it was found that offering strategies such as swimming, gym access, and psychological support by the university contributes to SEC development. These activities improve physical and mental well-being, which in turn contribute to better academic performance and a greater ability to concentrate and solve problems. Therapy can help students develop self-awareness, self-control, empathy, and resilience, all of which are crucial aspects of SECs [18].

Finally, challenges related to strategies for developing SECs were evidenced, including gaps in the curriculum matrix and teacher unpreparedness. A possible reason for this curricular gap may be attributed to resistance to changes in educational structures. Higher Education Institutions often face significant challenges in integrating new subjects and reformulating curricula. This can involve bureaucratic processes, the need for continuous teacher training, and the adaptation of pedagogical methods to address socio-emotional competencies effectively [5,6,7,30].

Another factor to consider is the reflection on the predominant view of what constitutes the “essential” in nursing education. The pressure to ensure graduates acquire a vast set of technical and practical knowledge can result in an overloaded curriculum, making it difficult to find space for additional subjects. There is also the idea that SECs are developed naturally through practical experience and social interaction, not necessarily through formal instruction.

The growing awareness of the importance of the mental and emotional well-being of health professionals has begun to change this perspective. Studies show that nursing professionals with strong socio-emotional competencies have better mental health outcomes, lower burnout incidence, and provide better quality patient care. These findings are pressuring educational institutions to reconsider and adapt their curricula to include training and strategies aimed at developing these competencies [6,7,8,9,31].

Moreover, teacher unpreparedness is a constant concern among participants. These data align with other studies on the topic. When directing our attention to studies addressing teachers’ emotional competence in the Brazilian context, we verify that, although scarce, the topic has been gradually occupying researchers’ attention [32,33]. In the international context, academic practices for developing emotional competencies are still limited. In the school environment, several factors can cause teachers’ emotional imbalance, ranging from external pressures to school indiscipline, violence, disrespect, discomfort, and illness, potentially leading to teachers leaving the work environment [14].

Research results point to the influence of emotional competence on the teaching–learning process dimensions, either in how the teacher manages their emotions and conflicts in the classroom or in the empathy with which they treat their students. Studies also indicate that teachers feel the need for more work focused on developing emotional competence in their initial and continuing education. This justifies our intent to investigate what the research points out about emotional competence in teachers’ education [32,33].

The paradigm shift to a more integral view of the individual represents a challenge for education, which can no longer be limited to academic knowledge. Preparing students for the challenges of complex reality demands a lot from teachers, who also need to be prepared to deal with emotions. Thus, creating a balanced, harmonious classroom environment favorable to the learning of values and disciplinary content has become a significant challenge and requires diverse knowledge and competencies from teachers, such as emotional competence [34].

Developing SECs in nursing students is essential to train professionals capable of providing humanized and high-quality care. To achieve this goal, several pedagogical strategies can be implemented in the nursing curriculum. These strategies, when integrated into the nursing curriculum, promote a holistic learning environment that goes beyond technical and scientific knowledge and involves a combination of theoretical and practical approaches that promote self-awareness, empathy, effective communication, emotion management, and resilience.

### Study Limitations

This research has the limitation of being conducted solely in a public HEI, not covering the analysis of other training centers. Additionally, this research was conducted exclusively with final-year nursing students. Therefore, it is important that further studies be conducted to encompass other educational institutions, beyond the already-graduated professionals or even other professional categories, to verify if there are differences and/or to determine the extent of the findings’ generalizability.

## 5. Conclusions

Addressing the study’s objective, several pedagogical strategies for developing socio-emotional competencies (SECs) and their main challenges were identified. The deficit in the approach to curricular subjects highlights the need for curriculum restructuring and further discussions on this topic.

Researching pedagogical strategies for developing SECs is essential because these competencies—such as empathy, emotional self-regulation, cooperation, and conflict resolution—are crucial for the well-being and personal and professional success of individuals. In an educational context, promoting the development of these skills can improve the learning environment, increase student engagement, and better prepare students to handle the challenges of everyday life. Furthermore, SECs are increasingly valued in the job market, where the ability to work in a team, communicate effectively, and manage emotions is vital. Research in this area helps identify effective pedagogical practices tailored to students’ needs, which can be implemented to promote holistic and balanced development. This contributes to forming more resilient, empathetic, and socially responsible individuals.

Additionally, research in this area contributes to building a body of knowledge that can guide educators and educational institutions in implementing innovative pedagogical practices. With concrete evidence on the benefits of certain strategies—such as realistic simulations, observation-based learning, tutoring, academic leagues, psychological support, physical activity, and mentoring—it is possible to justify and structure curricular changes that better meet students’ training needs. In turn, this improves not only the educational experience of students, but also their readiness to face the emotional and social challenges of the clinical environment.

The mental health and well-being of nursing students are also critical aspects that benefit from a pedagogical approach focused on socio-emotional development. Nursing professionals often face high levels of stress and burnout due to the emotional demands of their practice. Equipping students with socio-emotional skills during their training can help mitigate these negative effects, promoting greater resilience and self-care abilities, which are both essential for career sustainability and maintaining a healthy work environment.

## Figures and Tables

**Table 1:** Strategies for developing SECs according to their being offered by the curriculum matrix, the Nursing Department, and the university, and challenges related to developing these competencies.

	Offered by the Curriculum Matrix	Offered by the Department	Offered by the University
Strategies for developing SECs	Simulation; Assertive Communication; Welcoming; Teacher’s Posture; Discussion Circle; Case Discussion; Observational Learning (from model professionals).	Student Association; Tutoring; Scientific Initiation; Workshops provided by the post-graduate.	Gym; Swimming; Psychological Therapy.
Challenges associated with strategies for developing SECs	Subjects focused solely on the patient and not on professional posture; Scarcity of subjects addressing the topic; Lack of teaching didactics; Lack of teacher welcoming; Focus on traditional teaching methodologies.		

## Data Availability

Data were collected at the University of São Paulo, Ribeirão Preto School of Nursing. This is post-doctoral research that has not yet been published.

## Public Involvement Statement

Data were collected at the University of São Paulo, Ribeirão Preto School of Nursing. Participants were students from the listed university. All ethical precepts were upheld.

## Guidelines and Standards Statement

To prepare the study, the Consolidated Criteria for Reporting Qualitative Research (COREQ) guide from the Equator Network was used.
